# Clinical Studies with Large Non-Emetic Doses of Ipecacuanha: With a Contribution to the Therapeusis of Cholera

**Published:** 1875-05

**Authors:** Alfred A. Woodhull

**Affiliations:** Assistant Surgeon U. S. Army


### CLINICAL STUDIES WITH LARGE NON-EMETIC DOSES OF IPECACUANHA: With a Contribution to the Therapeusis of Cholera.

(Concluded, from page 28 of April number.)

By ALFRED A. WOODHULL, M.D., Assistant Surgeon U. S. Army.

*A Special Report to the Surgeon General U. S. Army, read before the Atlanta Academy of Medicine.*

This paper treats of ipecacuanha as a whole. Some of the later therapeutical experiments have been made with emetia, "pure" and "impure," which is certainly an active agent; but in whatever form administered it is usually, and often violently, emetic, and in excessive or repeated doses it is certainly dangerous. I have had no experience with it, but I greatly question whether the chemical mutilation to which the vegetable is subjected by its extraction does not radically destroy some essential quality. Although analysis seems to leave no room for any other active principle, it might be worth while to institute a series of careful experiments with the residuum after emetia is removed.†

I therefore regard *ipecacuanha as a peculiar but direct nervous stimulant, acting chiefly and probably entirely through the medium of the sympathetic system.*‡

† Dr. Squibb, of Brooklyn, informs me under date of 11th November, 1874, that "there is very much Ipecac now in the market that is of very doubtful character. It is a large size root, produced in the West Indies, is cheap, and is used either as a substitute or adulterant of the true Rio Ipecac. Perhaps half the powdered Ipecac sold may be made from this variety."

‡ Sunderlin, of Berlin, (*Handbuch der Speziellen Heilmettellehre,** 1825, ii., p. 38,) nearly fifty years ago taught that emetia exercised an exhausting stimulus over the eighth pair of nerves. (Duckworth, *op. cit.*, v., 1869, p. 221.)

The empirical fact remains, whatever explanation we may attach to it, that ipecacuanha is a valuable remedy in the sweats of phthisis, (and why not in others that are colliquative?) in passive hæmorrhages from the uterus, lungs, etc., in certain active hæmorrhages on the authority of Trousseau, in the collapse of cholera morbus, and in various forms of diarrhœa and dysentery. It does no violence to any known physiological or pathological fact to suppose that all these morbid states may be different manifestations of deranged sympathetic [ganglionic] action. For, as the ordinary malarial poisoning shows itself sometimes in an intermittent, sometimes in a remittent, and again in a neuralgia or hemi-crania—all disappearing under the influence of quinine—so, these various just-mentioned, but not necessarily identical, conditions may admit a common causative chain binding them together and leading them to yield to the same element of cure. That is to say, we may look upon those classes of disease, including serous diarrhœas and cholera infantum, and also the profuse cold (or passive) perspirations of consumption, cholera and fright, as brought into existence by various, and it may be dissimilar, direct or reflex disturbances of the ganglionic system. These may be generated by any impression, moral or physical, operating upon a nerve-centre. Where it is from irritating ingesta, the causes operate as long as the foreign bodies remain: where it is from an emotion, like fear, the effect passes off as equanimity returns. It is unnecessary, by multiplying illustrations, to trace the family likeness through all its gradations to the fully-developed type of the profoundest sympathetic disturbance.

And I here interject a paragraph, framed after this paper was written, that at least abuts upon, if it may not actually be an extension of, our theme. Dr. Wilson Fox, in his work on Diseases of the Stomach, now re-printing in the *Medical News and Library*, treating of hæmorrhage from that viscus, writes (Oct., 1874, pp. 248-9,) to this effect: in some of those capillary hæmorrhages which arise from congestion, there is probably, in addition to the congestion, "some alteration in the coats of the capillaries * *." "In the same manner are probably produced the hæmorrhages of yellow fever, and of other [?] malignant intermittents, as also those which occur in relapsing fever, typhus fever, cholera, purpura, scurvy and hæmorrhagic variola. In other cases, though probably referable to the same source, its mode of origin is less explicable; as when it follows severe surgical operations or blows upon the back or epigastrium, or even a remarkable case reported by Empis, where the invasion of tubercular meningitis was associated with uncontrollable vomiting with hæmatemesis." I have no desire to appear wise above what is written, but it appears to me that a profound sympathetic [ganglionic] derangement is an essential factor in all these conditions. The alterations in the blood that may occur in the various diseases named are of course to be reckoned, but the want of nervous power [paralysis, shock,] is most probably an important element—and that, to my mind, is the only rational explanation of the "less explicable" cases noted.† Why sometimes the outpouring is serous, sometimes hæmatoidal, and sometimes of pure blood, our present knowledge is not sufficiently refined to demonstrate.

Now cholera is *sui generis* only in the peculiarity of its reproductive power. Professor Stille, in a comprehensive lecture on this disease, asks (*Phila. Med. Times*, iii., p. 648,) "In what does sporadic cholera differ from malignant epidemic cholera?" and answers, "only in its cause and its degree. Its mechanism is the same," and that the epidemic "differs from the sporadic form chiefly by the intensity of its cause, the gravity of its symptoms and the nature of the special cause that produces it." Meigs and Pepper (*op. cit.*, pp. 378–399,) make a strong argument for the practical identity, saving the feature of self-propagation, of

---

† I would account for the case of ipecac-cured dysentery, occurring after an operation for hernia, in St. Bartholomew's Hospital (*St. B. Rep.*, vii., 117,) in this manner, i.e., by the nervous shock: and I suppose that the diarrhœas of the tuberculous that have been cured by ipecacuanha depended not on ulceration, but on irritation, perhaps from morbid deposits.

I suppose also that "the puerperal state," in the complications of which unquestionably ipecacuanha has been used with advantage, especially in France, has its peculiarity in the shocked and susceptible condition in which the abdominal sympathetic is placed as the result of the profound uterine disturbance lately undergone.

And in like manner the purest form of dysentery is that induced by climatic or epidemic influences, and not that caused mechanically (by irritating ingesta). The occurrence of bloody stools, often regarded as evidence of inflammation, is not such proof. And it outrages all analogy to suppose that a violent form of such disturbance yields at once to a medicine whose general antiphlogistic properties are no better proven than those of the drug in question. Is it not more reasonable to regard *dysentery at its inception as the manifestation of a peculiar ganglionic intoxication, and to consider the intestinal inflammation, with its consecutive ulceration, a result of the malady, but not the primary or radical affection?*

cholera infantum with the epidemic disease.† Dr. Da Costa reports (*Am. Jour. Med. Sci.*, no. cxv., 1869, p. 124,) a case of sporadic cholera with intestinal lesions found at the autopsy identical with those of epidemic cholera, although in life the discharges were not similar nor were there cramps. Dr. Edward Goodeve says (*Reynolds' System*, i., p. 172,) "It must be granted that symptoms similar to collapse may be produced by poisons without any purging. I have seen people under the influence of malarious poison in Calcutta lie for hours as cold and pulseless and as embarrassed in breathing as in cholera." As once before quoted in this paper, the "rice-water flux * * may occur also in other cases in which, as in cholera, there is a neuro-paralytic condition of the digestive canal" (Sedgwick, *Lancet*, Dec., 1871, p. 644). And the large number of cases cited by Mr. Sedgwick in his Analogy show one or another pathological phase of the pestilence duplicated in some other affection.

But there is more than a casual or accidental relation between cholera and certain other so-called blood diseases, and particularly are septic cholera from poisonous gases and epidemic cholera closely allied. Singularly, it has been suggested‡ that cholera and dysentery are antagonistic; but really one tends to increase the liability to and the danger of the other, and the latter is a not infrequent sequel to the more dreaded disease. And in attempting to develope this feature of the case I trust that I may not seem to be pressing the doctrine of the correlation of diseases too far, nor may I, by an indiscreet advocacy, bring ridicule upon the powers of a valuable medicine. Just as our scientific vision gains a wider range, do we better see the alliances that seemingly different conditions sustain with each other. If such a figure may be tolerated in a serious paper, I would say that, possibly, cholera, dysentery and the periodic fevers are a triune dæmon, each of whose faces represents a peculiar influence to be propitiated by especial offerings. We may never detect the real essence of this malignant trinity, and may never weave a spell that shall completely exorcise it: but all things are possible to the patient and the daring, and it is a worthy ambition to unravel such a secret and to compose such an incantation.

† Polichronie, (*op. cit.*, p. 33,) writing of the different forms of diarrhœa in children, speaks of "*le cholera infantile, qu'il est souvent presque impossible de distinguer du veritable cholera asiatique.*"

‡ I have met with this opinion in my reading, but have mislaid the reference.

There are certainly some very remarkable similarities in the apparent origin if not in the outward expression of these diseases;† and, speaking generally, not universally, we may include cholera morbus or the sporadic form under the wider category; just as the ordinary catarrhal dysentery is but a variety of the epidemic disease. In both, the contagious or catholic estates embrace the subdivisions. Niemeyer distinctly asserts that epidemic dysentery is closely allied to cholera (*op. cit.*, ii., p. 667,) and points out some marked constitutional similarities. Dr. Woodson noted in connection with his series of dysenteric cases near Gallatin, Tenn., treated by ipecacuanha in the summer of 1873, (*ante*,) the interesting fact that "previous to their outbreak a diarrhœal tendency had been observed in the same district which if not a consequence of was at least coincident with the prevalence of epidemic cholera at Nashville and Gallatin." It is well known that by a number of very respectable medical men it has always been held that Asiatic cholera is essentially a malarial disease; that it is only a virulent modification of the ordinary swamp fevers. And, as Niemeyer says, (*op. cit.*, ii., p. 622,) "we do not know why, but great epidemics of intermittent have often preceded epidemics of Asiatic cholera. In hot countries cholera and intermittent and oftener dysentery and intermittent frequently prevail at the same time;" and later (p. 637), speaking of the course of the severer forms of remittent fever, he states that in some cases there are "symptoms of cholera or dysentery." "Hersch [Hirsch] says it is a well-known fact, that malarial fever has preceded outbreaks of cholera, not only in single places or particular regions, but in an almost pandemic distribution, and there is every reason to believe that mala-

† While this paper is passing through the press I have received the British *Army Medical Department Reports* for 1872, (London, 1875, pp., 557,) and find that Deputy Surgeon-General Munro therein (pp. 266-274,) expresses the opinion that remittent, intermittent, congestive remittent [pernicious], cholera, yellow fever and heat apoplexy [insolatio] are different degrees of paralysis of the sympathetic nervous system, and that quinine is the remedy most to be relied on in all of them. He does not include dysentery in the group, and he denies the existence of malaria.

And I find that I have overlooked until the last moment the comprehensive remark of Professor Maclean who, speaking of malaria, in which he is a firm believer, says (Reynolds' *System*, i., p. 52,) "It is the cause of intermittent and remittent fevers, and their sequels: it 'underlies' the cause of dysentery and cholera;" etc. This was printed in 1866, and fairly coincides with the views expressed in the text as well as far antedates them.

ria and cholera devastate the same ground." (Peters, *op. cit.*, p. 127). Now (Aitken, 1st Am. ed., i., p. 381,) "it may be stated, as a general proposition, that there is no country where paludal fever exists in which dysentery is not an endemic and prevailing disease. * * This connection is so intimate that a given number of persons being exposed to the action of paludal miasmata—as, for example, a boat's crew sent ashore in a tropical climate—the probabilities are, that of the men returning on board, part will be seized with dysentery and part with remittent fever. Paludal fever and dysentery, moreover, are not only conjoined in locality, but they often co-exist, precede or follow each other in the same individual, so that the fever frequently ends in dysentery and the dysentery in remittent fever." Further, M. Marey (*Gaz. Hebdom.*,* Nov. 24 and Dec. 1, 1865; by Burral, *op. cit.*, p. 137,) finds a resemblance "between cholera and paroxysmal fevers, which latter he considers as under the control of the vaso-motor system of nerves." Now the vaso-motors themselves, although not derived from, are in great measure influenced by the sympathetic. And in this connection there is invited study of a valuable paper by Dr. Enrique M. Estrazulas, in the *American Journal of the Medical Sciences* for July, 1873, (no. cxxxi., p. 74,) clearly detailing the spontaneous origin of epidemic cholera in the camps of the allied and the opposing armies at and near Estero Bellaco, at the junction of the Paraguay and Parana rivers, in 1866, during the Paraguayan war, and the circumstances under which it occurred. He makes no claim for the purely paludal origin of the disease, but the facts presented distinctly show, I think, that for this pestilence along with the dysentery and malarial fevers that ravaged the forces, there could be found, in the general subtropical decomposition that prevailed, a common factor of production.†

And if we may speculate upon etiological affinities, we may certainly dream over therapeutical resemblances. There are few drugs limited to a single or specific action. The more familiar that we become with the materia medica, the more clearly do we see that classes of remedies operate in the same general manner, that few medicines are limited in their usefulness to any solitary pathological indication, and that individual remedies often have very varied action. It is in this very province, the action of medicines, that there is the least accurate knowledge. Even the

† See also an article on *Cholera: Does it originate de novo?* by Dr. W. Alston. (*N. Y. Med. Jour.*, xxi., 2, Feb., 1875, p. 126.)

so-called specific, quinine, has other powers than simple anti-periodicity, and these come into operation as necessities vary. We are justified in supposing that it is thus with ipecacuanha. As a specific it may be an emetic, but it has other applications. Final analysis may ultimately prove that the same drug always exerts the same kind of influence; but the conditions under which it is exerted so fluctuate that we are authorized in calling the modified manifestations different powers. Nor need we multiply illustrations of the unexpected modifications that size of dose and and condition of patient induce. Twenty grains of calomel will produce no annoyance, when a fourth of that amount would be painful and irritating; half an ounce of the tincture of digitalis will restore strength to the trembling pulse in delirium tremens, when half a drachm would cause the heart to flutter more wildly. The tolerance of opium in peritonitis, and of alcohol in snake-bite, are well known. The frequent tolerance of ipecacuanha in dysentery is established; its rejection in cholera is not proven, and is by no means necessary.

If the pathological and therapeutical views here expressed are well-founded, we are not to look for one drug as a cholera-specific or antidote—a neutralizer, as vaccination antagonizes variola—but we will find that various ganglionic and vaso-motor stimuli may profitably be employed in cholera, and that ipecacuanha may be used in other disturbances of the sympathetic and vaso-motor systems, as indeed I believe has already been illustrated. Just where its maximum of power with the minimum of resistance are to be found, is yet entirely unsettled. In confirmation of this general view is explained the action of atropia, which is understood to cause contraction of the capillaries, and which has been used hypodermically with a certain degree of success.† But the most valuable therapeutic contribution hitherto made to this subject is the employment of the bromide of potassium, based on the pathological hypothesis herein expressed. Thus, in 1873, Dr. William Pepper, on theoretical grounds, suggested (*Phila. Med. Times*, iii., pp. 651, 742,) its intravenous injection in solution. I do not know that this has been put in practice. Dr. Pepper, however, has been anticipated in its general use by Dr. James Begbie, of Scotland, (*Edin. Jour.*, 1866, xii., pp. 488, 490,) who, from identical reasoning, recommended it, and on whose recom-

† Ergot, whose action is of the same general character, has also been used, but not very successfully.

mendation it was used in the Leith and Edinburgh Cholera Hospitals. Dr. Begbie says that "although not possessing the properties of an antidote to the poison of cholera, though not a specific to the shock of this terrible disease, [it] has certainly stript it of some of its terrors." This view has received an independent but strong support by a series of cases published by Dr. Salvator Caro, (*Med. Record*, iv., p. 195,) in 1869. That paper gives in detail twenty out of one hundred and sixty-three cases, running through all the morbid states, from a simple serous diarrhœa to cholera infantum, dysentery and septic cholera, and embracing young and old, where it was successfully used. And the conclusions of one of the latest investigators, Dr. Robert Amory, (1872,) give abundant theoretical confirmation. He has satisfied himself, (*Phila. Med. Times*, ii., p. 335,) that: "The effects of the drug are produced by its direct action upon the blood-vessels or the vaso-motor system which controls the contraction of those vessels, which explanation may account for all the physiological or therapeutical conditions brought about by the exhibition of the drug."†

But recurring to M. Marey's opinion, that both cholera and the periodic fevers are due to vaso-motor disturbances, we find that Dr. John Murray, late Inspector General of Hospitals, Bengal, in his *Observations on the Pathology and Treatment of Cholera*,* (1874,) strongly advises in the premonitory stage the use of two-grain doses of quinine three times a day (Review in *Phila. Med. Times*, iv., 1874, p. 636); and, on the other hand, we are reminded that the emetic action of ipecacuanha, under the idea of producing 'a shock' or of 'breaking up the habit,' (an explanation savoring more of mediæval mysticism than of modern therapeutics,) has been frequently invoked in the treatment of an ague, especially when obstinate. And in the *Indian Medical Gazette** for June, 1872, (*Phila. Med. Times*, ii., p. 416,) Udhoy Chand Dutt, a civil medical officer in India, reports the cure of seventy-four out of seventy-six cases of intermittent, in from three to five days, by the administration of minute doses of ipecacuanha.‡ It is perfectly conceivable that the sympathetic may be affected in a way to give rise to the intermittent phenomena of miasmatic

† H. C. Wood considers (*op. cit.*, pp. 281, 283,) "no decisive proofs have, however, yet been offered of the truth of this favorite dogma."

‡ It was formerly an English, and is yet a common Italian, practice to administer an emetic of ipecacuanha at the beginning of an intermittent at-

[paludal] disease, although it does not follow that we can explicitly describe or accurately paint the actual histological conditions involved, and that moderate [or 'alterative'] doses may correct that state. Such an hypothesis explains the pseudo-choleraic collapse of certain grave forms of the disease, and encourages in them the non-emetic employment of the drug. For the absence of the choleraic discharge does not militate against the idea that the same division of the nervous system may be deranged with different manifestations.

And as, returning to the pathology of dysentery, which in grand outline resembles cholera and where the general fever and so-called inflammatory condition of the first stage are not proportional to the suffering, we find that when ipecacuanha is properly given before organic changes [ulcerations] occur relief is speedy, so we are bound to consider that the drug in some way, directly or indirectly, antagonizes the toxic principle, allowing health to return. If anything in practice is certain, it is that bleeding and calomel will not abort acute dysentery and that ipecacuanha fairly abolishes it. We have at the least, therefore, a fair presumption in our favor when we anticipate that the hydrorrhagia of the more alarming pestilence may cease † as promptly as the smaller and more hæmatoic discharges of the commoner disease. But it requires faith and a certain kind of courage to administer to a patient, already sadly vomiting, what for two hundred years has been the type of an emetic. But, used with care, I am confident that it checks that symptom if it depends on no extrinsic cause. It is the first step that counts; that taken, the rest are easy. Authentic empirical illustrations of its

---

tack; and although the French think that it is only useful by relieving gastric embarrassment, many claim that it has a febrifuge action analagous to that of quinine. Polichronie (*op. cit.*, p. 24,) regards it of sufficient interest to merit renewed research.

NOTE.—Between the time of writing this paper and that of printing this portion of it, I have experimented with non-nauseating doses of ipecacuanha in intermittents, and have found in more than twenty consecutive cases that it controlled the disease as promptly as quinine would have done. I hope to be able soon to publish the details. Meanwhile clinical studies, with careful notings of pulse and temperature, could easily be made, and would probably compensate for the trouble.

† It is to be remembered that, during this very summer [1874] cholera infantum, so analagous to Asiatic cholera in its manifestations, yielded promptly to ipecacuanha in the hands of MM. Chouppe and Huchard (*vide supra.*)

power dot medical records for at least sixty years. Give ipecacuanha freely but cautiously—cautiously does not mean timidly—in the vomiting of exhaustion, and it will arrest it. There is no invariable human formula, but anti-emesis quite as often as emesis will be the expression of its function.

There are two additional fragments that I desire to introduce into this mosaic before it leaves my hands, imperfect and perhaps unintelligible to others as it may even then remain.

The first concerns the condition of the gall-bladder in collapse and the absence and reappearance of bilious stools. An essential, if not the pathognomonic, symptom of the disease, notwithstanding its misnomer, is the absence, not the flow, of bile; and a large section of the profession has sedulously occupied itself, by the employment of calomel and other presumed cholagogues, in the attempt to re-establish that discharge; for the reappearance of bilious stools is universally hailed as a sign of convalescence. Now the gall-bladder is generally found filled in collapse, (notwithstanding that vomiting is supposed to mechanically force out its contents,) and the retention of bile is only the sign, not the cause, of the disease. Undoubtedly bile flows because convalescence begins; health does not return because bile flows. And we may readily understand why this is so when we remember that the muscular tissue of the gall-bladder is unstriped, and is under the nervous control of the sympathetic. If that nerve is paralyzed this receptacle does not give vent to its contents; when the sympathetic reasserts its power the discharge reappears.

The second is the following. Mr. Sedgwick, (*Lancet*, Dec., 1871, p. 646,) in a sentence opposing the purgatives of the Johnsonian teaching, uses these words: "a careful and scientific investigation of the stage of convalescence, especially with reference to the occurrence of temporary glycosuria," etc. From this I do not understand whether he refers to temporary glycosuria as a well-known and admitted fact, or means to suggest that it may occur and should be looked for. I have found no other reference to its existence in the authorities that I have been able to consult. For myself, I do not know whether sugar is present in the urine that begins to appear with the establishment of reaction, but, if this should be the case, it seems to me susceptible of an explanation, curious from the nice interplay of somewhat complicated conditions and affording another argument for the employment of the drug. (It may seem presumptuous to seriously ask attention to so much that is supposititious. But the suggestion may at least lead to investigation by those better prepared for investigation.) We know, or at least we believe, that diabetes depends on the dilatation of the capillaries of, and on the consequently more rapid circulation of blood through, the liver, and that it follows the paralysis or exhaustion of that part of the sympathetic that supplies it. Professor Cyon (*Brit. Med. Jour.,** Dec. 23, 1871, quoted in *Phil. Med. Times*, ii., p. 196,) has shown that the fibres composing the annulus of Vieussens particularly preside over the hepatic circulation, and that their irritation induces the diabetic condition. But if the entire sympathetic is cut or paralyzed diabetes does not occur, because "those parts of the nervous system contain the vaso-motor fibres for the vessels of the intestines: and when they are cut, the vessels dilate, and blood accumulates in them to such an enormous extent that there is either too little blood remaining, or it is under too low pressure for the circulation in the liver to become increased above its normal, even though its vessels be dilated." We know, however, that the liver is found gorged with blood when death occurs in collapse, or, that is to say, when its sympathetic fibres are paralyzed. We may therefore naturally infer that when reaction begins and the circulation tends to recover its usual tone, more blood than usual passes through the liver under the combined effect of the partly dilated vessels and the increased force of the circulation, and we might therefore look then for the temporary glycosuria that could occur neither in the profound stage nor when the health and the normal circulation are restored. And this pathological condition gives support to the therapeutic view here advocated, when we remember that, as long ago as 1862, Pecholier announced (H. C. Wood, *op. cit.*, p. 364; fr. *Gaz. Med.,**) "that in animals killed by it [ipecacuanha] no hepatic glucose can be found."† The inference of course may be drawn that it suspends the glycogenic function because it acts upon the vaso-motors (through the sympathetic) in directly the reverse manner in which traumatic injury or cholera poison is active. The disease paralyses the nerves and dilates the vessels; the drug stimulates the nerves and contracts the vessels.

† Pecholier, (*op. cit.*, p. 40,) "*nous avons constate des efforts des vomissements, * * * la disparition du sucre dans le foie.*"

These explanations of the two conditions just described satisfy my own mind and, so far as I am aware, have never heretofore been published.

As a matter of course, the whole materia medica has been ransacked for a cure for the pestilence that has girdled and devastated the globe. And in these trials so common a drug as ipecacuanha has been frequently employed, but generally, if not universally, as an emetic. Dr. George Johnson has used it in his eliminative practice (and it would be interesting to analyze his statistics with a view to observe if the so-called tolerance was established, and whether there was any observable connection between the degree of his success and the amount of this medicine that was retained). Peters writes (*op. cit.*, p. 139,) "Waring says, the mortality has been very large under its use when given in full emetic doses. Others say it has been given successfully in five or ten grain doses every five or ten minutes. It causes violent attempts at vomiting, but after three or four doses tolerance is established. In the Paris hospitals, in 1865, ten to twenty grains were given whenever there was much vomiting." I have not been able to discover the originals or the particulars of any of the three statements just cited, but they all seem to refer to its emetic use.† Waring, however, after reprobating its emetic use as an eliminative, does say (*Prac. Therap.*, p. 361); "A far more promising practice is to administer it in very small, often-repeated doses, in the manner employed in hæmorrhages by Mr. Trenor. In the latter affections, even when a state of collapse supervened, the vital powers recovered themselves in a striking manner under the use of ipecacuanha; and the same remedy seems to merit a trial in cholera, even in the stage of collapse; the many points of similarity between cholera and profuse hæmorrhage would alone suggest its probable utility. The more recently ascertained facts with regard to the power of minute doses to arrest vomiting are

† M. Decugis writes (*loc cit.*, p. 40.) "Cholera.—At its first appearance, in 1832, the physicians, struck by its resemblance to dysentery, proposed ipecac. M. Grisolle believed the remedy to be a specific (*crut a la specificite de ce medicament*) against the cholera; but in 1849 he was obliged to recognize the slight utility of the administration of that substance. M. Briguet employed it also at the Charite with as little success: for ourselves, we have known, during the epidemic that scourged Toulon in 1865, that ipecac did not more than other remedies succeed in assuaging the grievous attacks of that terrible disease."

strongly in favor of its probable efficiency."† If it is allowable to discuss hypothetical conditions in the absence of practical demonstration, I should say that one of the differences between the hæmorrhagic and choleraic conditions is, that in the former the capillary lesion is, so to speak, passive, the result of exhaustion; while in the latter it is active, being impressed by the positive toxic element: and that, while small doses might be trusted to restore the capillary tone in the one or negative condition, unless we embrace the homœopathic doctrine of attenuations and potencies, we must use larger quantities to antagonize the active morbific influence. It is my belief that emesis is influenced less by the size of the dose than by the manner of its administration. "The doses given by Mr. Trenor varied from gr. j.–ij. every fifteen or thirty minutes until nausea was felt," (Waring, *op. cit.*, p. 360,) while in a large series of cases published by Dr. Samuel Pye, "The average quantity which he gave was only two grains, yet it generally produced vomiting three or four times, and sometimes oftener." (Stille, *op. cit.*, ii., p. 391.) On the other hand, I have repeatedly given twenty-five grains without inducing vomiting, and one and two drachms have been similarly administered in East Indian practice.

A corroborative suggestion to the therapeusis proposed in this article I have found in a paper by George Barnard, Esq., Surgeon 6th B. L. I., (*Am. Jour. Med. Sci.*, no. cxiii., Jan., 1869, p. 246,) who takes the ground that cholera is practically an intense inflammation of the mucous membrane, and advocates its treatment by grain doses of tartar emetic every fifteen minutes until vomiting ceases, and further says: "30 grains of antimony's ally, ipecacuanha, will have the same effect given in the same way every quarter of an hour. See Docker's case of forty grains ipecac in advanced collapse, (*Lancet*) and 392 cases by Dr. Carl Muller, Vienna." I should hardly be willing to subscribe to the pathology advanced or to the antimonial treatment advocated, but the implication that ipecacuanha has been actually employed in this manner is extremely interesting. The references are so

† Of this, although it occupies a prominent place in his valuable article on the drug in question, it is proper to observe that I was unaware when I used it in large doses in the cases of cholera morbus, and when it occurred to me that it might be available in epidemic disease. It may also be noted that Dr. Waring regards ipecacuanha as a sedative, (*op. cit.*, p. 356,) and offers no rationale for its presumed action in such cases, except indirectly and by implication.

indefinite that I have been unable to verify them; but if it should prove that three hundred and ninety-two *successful* cases of the use of ipecacuanha in large doses are on record, that ought to settle the question empirically, whatever may be ultimately demonstrated as its mode of operation.

I earnestly entreat of those who may be tempted by anything that has been said in these pages to use large doses of ipecacuanha in the diseases discussed, that they will carefully adhere to the non-emetic method. Without doubt untold suffering has been endured during the past two centuries from the gradual abandonment of the remedy after its triumphant introduction into Europe and before its recent revival in Asia—a disuse for which this disagreeable and wholly unnecessary concomitant is chiefly responsible. In practical medicine the least things are sometimes important.

I neither have the opportunity nor claim the ability to prepare an exhaustive essay upon these interesting subjects, but I have made this paper as complete as my means would allow, knowingly omitting nothing *pro* or *contra*. I have preferred to err on the side of prolixity, by actually quoting the authorities and carefully explaining my own meaning, than to be charged with misrepresentation or assumption as to the views of others, or with ambiguity and cloudiness upon my own part. And I have entered every reference as I have consulted it. I trust that I may not be charged with that vaulting ambition which o'erleaps itself, in the choice of a subject. The subject indeed forced itself upon me, and being present, I have sought to treat it honestly and fairly, with a simple desire to increase the means of relieving human suffering.

If, as I sometimes hope, the work that the theoretical part of this paper represents has any value, it is chiefly due to those skilful and earnest laborers in the domain of science who have collected the material and have unselfishly offered it for the public good. I have merely selected certain cuttings, and have drawn them into relationship. The little portal that I have built I hope may prove a minor entrance to the great cathedral of the common weal. It may be but a doorway to some subterranean or useless gallery, or at best be fit only for transformation into a fantastic gargoyle to carry off the waste water of the scientific skies. Should it be so, the material has not been damaged and it can easily be re-wrought. If happily the former, but little praise belongs to the lucky designer who has stumbled upon the carefully-hewn and generously-given blocks.

McPherson Barracks, Atlanta, Ga.

---

* The references marked with an asterisk (*) are derived at second-hand. Those not so marked I have persona'ly examined.

Note.—This paper, prepared as a special report to the Surgeon General of the Army, was completed early in October, 1874. It was forwarded to that officer, and the retained copy was read before the Atlanta Academy of Medicine 19th and 26th of that month. While transcribing it for the press, Dr. Duckworth's papers and the original French documents referred to fell into my hands, requiring certain interpellations and additional notes and delaying the manuscript until this date. I believe that no essential fact in this new matter has been overlooked; and I am glad to say that I have detected nothing requiring a change in my previously expressed opinions, while some apparent confirmations have been discovered.

28th *November*, 1874.

---

